# Proposal of Critical Nutrient Levels in Soil and Citrus Leaves Using the Boundary Line Method

**DOI:** 10.3390/plants14121764

**Published:** 2025-06-09

**Authors:** Antonio João de Lima Neto, Amanda Veridiana Krug, Jean Michel Moura-Bueno, Danilo Eduardo Rozane, William Natale, Jacson Hindersmann, Ana Luiza Lima Marques, Lincon Oliveira Stefanello, Daniéle Gonçalves Papalia, Gustavo Brunetto

**Affiliations:** 1 Department of Plant Science, Federal University of Ceará (UFC), Fortaleza 60356-000, CE, Brazil; antonio.joao@ufc.br; 2 Department of Soil Science, Federal University of Santa Maria (UFSM), Santa Maria 97105-900, RS, Brazil; krug.amanda@hotmail.com (A.V.K.); jacsonjh7@gmail.com (J.H.); marquesluizalima@gmail.com (A.L.L.M.); danipapalia@hotmail.com (D.G.P.); brunetto.gustavo@gmail.com (G.B.); 3 Department of Agronomy and Natural Resources, São Paulo State University (UNESP), Registro 11900-000, SP, Brazil; danilo.rozane@unesp.br; 4 Brazilian Agricultural Research Corporation (EMBRAPA—Agroindustry Tropical), Fortaleza 60511-110, CE, Brazil; natale@ufc.br; 5 Department of Agronomy, Federal Technological University of Parana, Santa Helena 85892-000, PR, Brazil; lincono@utfpr.edu.br

**Keywords:** fertilization in orchards, soil fertility in orchards, leaf nutrient sufficiency ranges, rational use of fertilizers

## Abstract

Establishing critical levels (CLs) and sufficiency ranges (SRs) for nutrients improves fertilizer recommendations and supports citrus yield and fruit quality. The objective of this study was to establish CLs, soil fertility classes, and leaf nutrient SRs for citrus. This study used data on the yield and nutrients of the soil and leaves, collected from 2016 to 2021, of commercial orange (*Citrus sinensis*) and tangerine (*Citrus deliciosa*) orchards in the Southwest and Metropolitan regions of the state of Rio Grande do Sul, in southern Brazil. The yield data were related to the soil attributes/leaf nutrient contents. From the models obtained from this relationship, soil fertility classes and leaf sufficiency ranges were established using the boundary line (BL) method. The appropriate classes are 5.1–5.6 for pH, 1.0–1.4% for OM, 65.8–129.0 mg dm^−3^ for P, 161.4–326.0 mg dm^−3^ for K, 0.9–1.4 cmol_c_ dm^−3^ for Ca, 0.22–0.34, cmol_c_ dm^−3^ for Mg, 1.9–2.9 cmol_c_ dm^−3^ for SB, 4.5–5.8 cmol_c_ dm^−3^ for CEC, and 40.6–53.2% for V. The appropriate ranges of leaf contents were as follows: 19.1–22.7 g kg^−1^ of N, 0.8–1.3 g kg^−1^ of P, 7.8–11.3 g kg^−1^ of K, 20.9–28.4 g kg^−1^ of Ca, 2.0–3.3 g kg^−1^ of Mg, 2.0–3.0 g kg^−1^ of S, 88.8–127.5 mg kg^−1^ of B, 28.3–73.6 mg kg^−1^ of Cu, 74.3–122.5 mg kg^−1^ of Fe, 55.7–89.3 mg kg^−1^ of Mn, and 10.9–15.6 mg kg^−1^ of Zn. The BL method made it possible to establish nutrient CLs using data from commercial orchards, which is not possible when using conventional approaches. The established norms will allow for a more precise definition of the real need for fertilizer application in citrus orchards.

## 1. Introduction

Citrus (*Citrus* spp.) is commercially grown in more than 140 countries in an area of approximately 10.6 million hectares, with a world production of 169.4 million tons, playing a key role in the agricultural economy. Brazil is the second largest producer in the world with an area of approximately 701.8 thousand hectares and a production volume of 20.5 million tons [1].

Citrus plants are predominantly cultivated in tropical and subtropical regions around the world, where soils, in general, are characterized by high acidity and low organic matter content, which are the main factors in limiting nutrients [2,3]. Because of this, the soil does not provide sufficient quantities of nutrients to meet the demand of citrus [4,5,6]. Therefore, it is necessary to use soil acidity correctives and fertilizers to meet the high nutritional demand of citrus plants, maintain high levels of yield, and produce fruits with excellent quality [7,8].

Liming and fertilization recommendations for citrus are based on the results of soil and leaf chemical analyses carried out periodically [8,9]. The efficient use of these tools requires the establishment of interpretation standards, which have been established in calibration studies carried out over several years and at various locations under a wide variation of soil and climate conditions [10,11,12]. However, the use of new scion and rootstock varieties in citriculture, as well as changes in the nutritional management of orchards, requires an update of these reference tables to interpret the results of the analyses [13,14].

Establishing diagnostic norms for soil fertility and the nutritional status of fruit-bearing plants through calibration studies takes a lot of time and is expensive [15]. As an alternative to classical calibration studies, parameters for interpreting soil fertility and plant nutritional status can be established using the boundary line (BL) method, which is a solution to the limitations of regional diagnosis [15,16,17]. This method has the advantage of using data from nutritional monitoring carried out by producers and consists of relating yield (dependent factor) to soil attributes/leaf content, fitting mathematical models to the points of the upper boundary that make it possible to establish the optimal value of the independent factor [15,16,17,18]. This method has been successfully used to establish adequate nutrient levels in soil and leaf tissues for various crops [15,17,18,19,20,21,22,23].

The objective of this study was to establish the critical and optimal levels of nutrients in the soil and leaf tissues of citrus for production areas, using the BL method, while showing why the traditional calibration methods are inadequate in this region.

## 2. Materials and Methods

### 2.1. Study Area

This study was carried out using a database with information on yield, soil fertility attributes, and leaf nutrient contents. These data were collected in non-irrigated commercial orchards of oranges (*Citrus sinensis*) and (*Citrus deliciosa*) tangerines, whose production is destined for fresh consumption, distributed in the Southwest and Metropolitan regions of Rio Grande do Sul, in the municipalities of Rosário do Sul, Pareci Novo, and Montenegro.

The data totaled 495 soil observations and 486 leaf observations, collected in the 2016/2017, 2017/2018, 2019/2020, and 2020/2021 seasons in citrus orchards, with ages ranging from 11 to 35 years, and planting density between 500 and 667 plants ha^−1^ (3 × 5 m; 3 × 6 m; 4 × 5 m; 5 × 3 m), consisting of three rootstocks (*Citrange troyer*, *Citrumelo swingle,* and *Porcirus trifoliata*) and thirteen varieties of scion (Caí, Cara Cara, Ellendale, Lane Late, Midnight, Montenegrina, Murcot, Nadorcott, Navelina, Ortanique, Pareci, Ponkan, and Salustiana). The orchards are planted in soils predominantly classified as Alfisols and Ultisols [24].

### 2.2. Soil, Leaf and Production Analyses

The soil was randomly collected in the 0–20 cm layer in homogeneous plots, in a total of twenty single subsamples to form a composite sample. The samples were collected in the crop row, where the fertilizers were applied. Then, the soil samples were air-dried, passed through a sieve (2 mm mesh), and chemically analyzed according to the methodology proposed by [25]. Soil pH was determined in water (soil: water ratio of 1:1), and available concentrations of P and K were extracted by Mehlich-1; exchangeable Ca, Mg, and Al were extracted with 1 mol L^−1^ KCl. P was determined by spectrophotometry (Bell Photonics, 1105, São Paulo, Brazil), K by flame photometry (Digimed, DM-62, São Paulo, Brazil), Ca and Mg by atomic absorption spectrometry (PerkinElmer—Analyst 200, Norwalk, CT, USA), and Al was titrated with 0.0125 mol L^−1^ NaOH. The sum of bases (SBs), cation exchange capacity at pH 7.0 (CEC pH_7.0_), aluminum saturation (Al), base saturation (V), and potential acidity (H + Al) were calculated. Total organic carbon content was determined by oxidation (Walkley–Black) and multiplied by 1.724 to estimate the soil organic matter (OM) contents.

Leaf collection was carried out on the same plants used for soil collection; the 3rd or 4th leaf (approximately six months of age) of the branch with terminal fruit of 2 to 4 cm in diameter was collected, for a total of four healthy and undamaged leaves per tree, with one in each quadrant and at the medium height of the plant [26] collected at the same phenological stage, according to the fruiting period of each variety.

After collection, the leaves were washed in distilled water, a detergent solution (0.1%), a hydrochloric acid solution (0.3%), and deionized water. Then, the plant material was dried in an oven at 65 °C for 48 to 96 h, and ground and analyzed for total nutrient contents according to the methodology described by [25]. A subsample of the tissue was subjected to sulfuric digestion to determine N content using a Kjeldahl steam-drag distiller. Another subsample was subjected to nitric-perchloric digestion and Ca, Mg, Cu, Zn, Fe, and Mn contents were determined in the extract by atomic absorption spectrophotometry (PerkinElmer—Analyst 200, Norwalk, CT, USA). P contents were determined by colorimetry [27] in a spectrophotometer (Bell Photonics, 1105, Brazil), and K contents were determined in a flame photometer (Digimed, DM-62, São Paulo, Brazil). B contents were extracted by burning 0.5 g of a plant tissue sample in a muffle furnace (600 °C for 1 h) and determined in a spectrophotometer (Bell Photonics, 1105, São Paulo, Brazil) [28,29].

The trees where soil and leaf samples were collected were marked and later used to obtain yield. To calculate the production, all the fruits of each tree were collected at the time of harvest for each variety, and the fruit boxes were weighed and multiplied by the number of plants per hectare.

### 2.3. Establishing Diagnostic Norms

The critical levels (CLs), soil fertility classes (FCs), and leaf nutrient sufficiency ranges (SRs) for citrus were established using the BL method as proposed by [17]. Before establishing the BLs, outliers were removed from the dataset using box-and-whisker diagrams in Statistica software version 12.5. Scatter plots were constructed relating the relative fruit yield (RFY) of each plot (*y*-axis) to the attributes of the soil fertility/leaf nutrient contents (*x*-axis) of the same plot [18]. Next, the *x*-axis was divided into 10–15 intervals and the highest point of each interval of the upper boundary was selected [22].

The data pairs (y,x) selected at the upper boundary (10 to 15 points) of the data cloud were used to establish mathematical models, chosen based on the best fit, significance, biological significance, and coefficient of determination (R^2^). These models were used to establish the critical levels, soil fertility classes, and leaf sufficiency ranges: Low (RFY < 70%), Medium (70% ≤ RFY < 90%), Adequate (90% ≤ RFY ≤ 100%), and High (RFY > 100%).

## 3. Results

### 3.1. Soil Fertility Attributes

The soils of commercial citrus plots, in general, are heterogeneous in terms of fertility attributes, with a coefficient of variation (CV) ranging between 12.6% and 91.1% (Table 1). The high CV values indicate that the soil fertility data of the evaluated areas present high variability. The soils of the orchards have a pH ranging from 4.1 to 7.1 and OM concentrations from 0.7 to 2.0%. The available P and K concentrations ranged from 1.2 to 2.66.6 mg dm^−3^ and from 16.0 to 590 mg dm^−3^, respectively. The Ca and Mg concentrations in the soil ranged from 0.2 to 4.2 cmol_c_ dm^−3^ and from 0.1 to 0.9 cmol_c_ dm^−3^, respectively. The sum of bases (SBs), cation exchange capacity (CEC), and base saturation (V) ranged from 0.5 to 5.2 cmol_c_ dm^−3^, from 1.7 to 9.2 cmol_c_ dm^−3^, and from 18.8 to 84.2%, respectively (Table 1).

### 3.2. Critical Levels and Soil Fertility Classes

**Figure 1:**
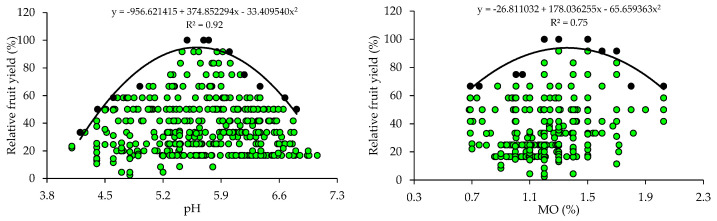

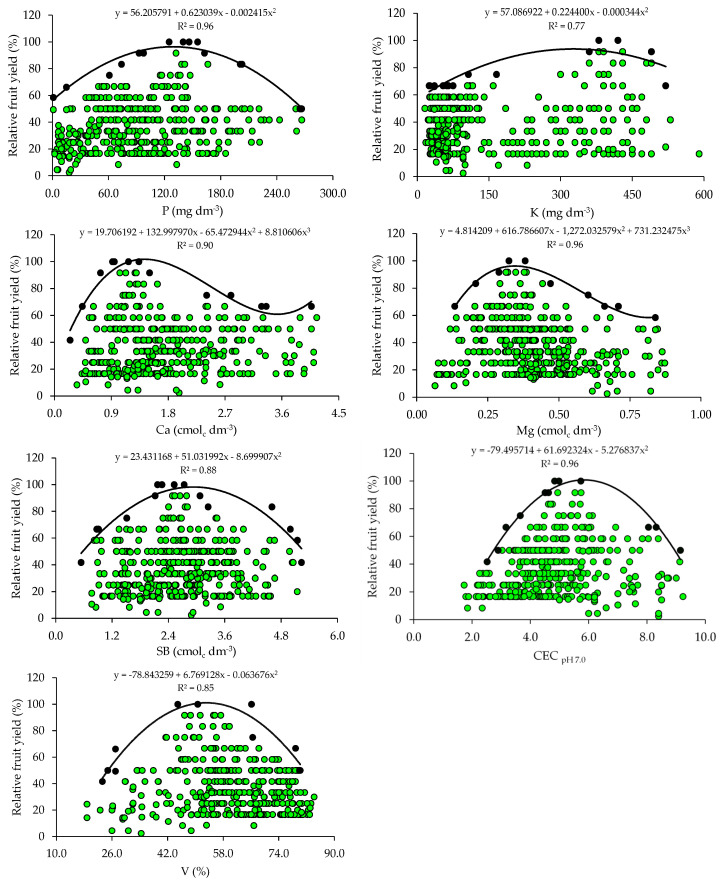
Scatter plots and boundary lines of the relationship between the relative fruit yield of citrus and soil hydrogen potential (pH), organic matter (OM), available phosphorus (P), available potassium (K), exchangeable calcium (Ca), exchangeable magnesium (Mg), sum of exchangeable bases (SB), cation exchange capacity (CEC), and base saturation (V) (0–20 cm).

The scatter plots show the relationship between the relative fruit yield of citrus and the attributes of soil fertility (Figure 1). These relationships allowed the fitting of mathematical models with coefficients of determination (R^2^) ranging from 0.75 to 0.96. The response curves obtained by the BL method indicate that the increase in pH and nutrient availability in the soil promoted yield gains up to a plateau, beyond which the excessive concentrations of nutrients are related to nutritional imbalances reflected in low yields.

From the equations obtained from the relationship between the relative fruit yield of citrus and the fertility attributes, the critical levels and soil fertility classes were established (Table 2). The adequate soil pH for crop growth and development ranged from 5.1 to 5.6 and the OM concentration ranged from 1.0 to 1.4. The P and K concentrations related to higher yields ranged from 65.8 to 129.0 mg dm^−3^ and from 161.4 to 326.0 mg dm^−3^, respectively. The Ca and Mg concentrations should be 0.9–1.4 cmol_c_ dm^−3^ and 0.22–0.34 cmol_c_ dm^−3^. Also, the soil must have a SBs ranging from 1.9 to 2.9 cmol_c_ dm^−3^, a CEC ranging from 4.5 to 5.8 cmol_c_ dm^−3^, and a V ranging from 40.6 to 53.2% (Table 2).

### 3.3. Leaf Nutrient Contents

Descriptive statistics of yield and the nutrient contents in citrus leaf tissues indicate that there is wide variation in the nutritional status of the plants in the orchards (Table 3). The yield of the orchards ranged from 6.0 to 244.8 kg plant^−1^, with an average of 85.9 kg plant^−1^. Leaf nitrogen (N) contents ranged from 14.9 to 32.2 g kg^−1^, P contents from 0.5 to 2.5 g kg^−1^, K contents from 4.2 to 19.6 g kg^−1^, Ca contents from 13.0 to 43.9 g kg^−1^, Mg contents from 0.8 to 6.5 g kg^−1^, and S contents from 0.7 to 5.8 g kg^−1^ (Table 3). The micronutrients B, Cu, Fe, Mn and Zn showed contents in the leaf tissues ranging from 13.4 to 224.1 g kg^−1^, 6.8 to 177.7 g kg^−1^, 36.7 to 243.7 g kg^−1^, 7.1 to 153.6 g kg^−1^, and 6.6 to 39.7 g kg^−1^, respectively.

### 3.4. Critical Levels and Leaf Sufficiency Ranges

**Figure 2:**
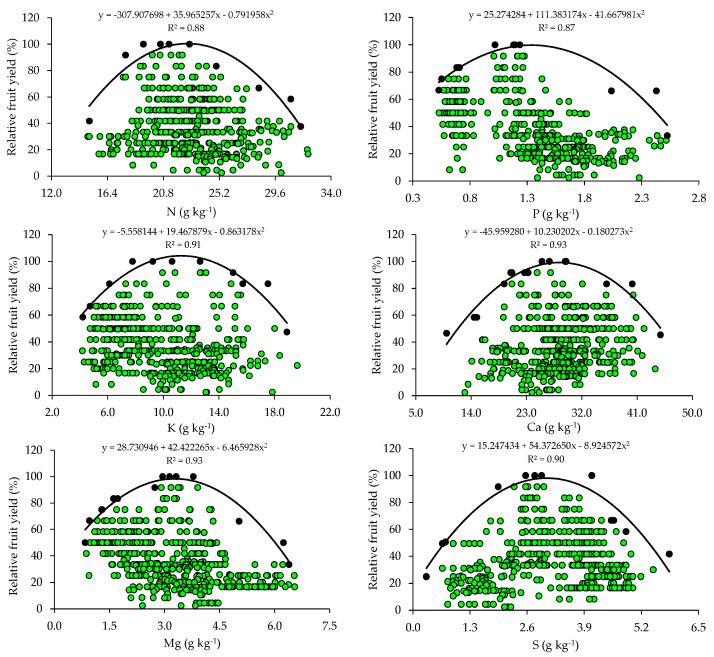
Scatter plots and boundary lines of the relationship between the relative fruit yield of citrus and the leaf contents of nitrogen (N), phosphorus (P), potassium (K), calcium (Ca), magnesium (Mg), and sulfur (S).

**Figure 3:**
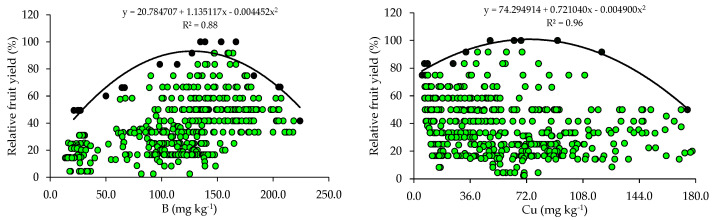

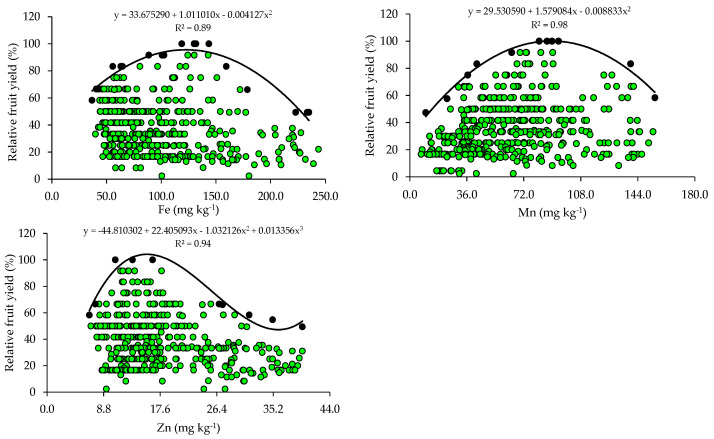
Scatter plots and boundary lines of the relationship between the relative fruit yield of citrus and the leaf contents of boron (B), copper (Cu), iron (Fe), manganese (Mn), and zinc (Zn).

The relationships between relative fruit yield and leaf nutrient contents are presented below (Figure 2 and Figure 3). Based on the relationships, the points of the upper boundary were selected, which allowed for the fitting of mathematical models with high coefficients of determination (R^2^), ranging from 0.87 to 0.97. These models were used to establish the critical levels and fertility classes of the nutrients in leaf tissue.

The critical levels and leaf sufficiency ranges of the nutrients were obtained from the response curves of the relationship between yield and leaf contents (Table 4). The appropriate contents for citrus are 19.1–22.7 g kg^−1^ of N, 0.8–1.3 g kg^−1^ of P, 7.8–11.3 g kg^−1^ of K, 20.9–28.4 g kg^−1^ of Ca, 2.0–3.3 g kg^−1^ of Mg, and 2.0–3.0 g kg^−1^ of S (Table 4). In the case of micronutrients, the ranges of adequate contents of B, Cu, Fe, Mn, and Zn are 88.8–127.5 mg kg^−1^, 28.3–73.6 mg kg^−1^, 74.3–122.5 mg kg^−1^, 55.7–89.3 mg kg^−1^, and 10.9–15.6 mg kg^−1^, respectively (Table 4).

## 4. Discussion

### 4.1. Relationships Between Soil Fertility and Yield

The citrus orchards evaluated show a wide variability of attributes related to soil fertility (Table 1). According to the classification proposed by [30], considering the coefficient of variation (CV), the variability is very high (CV > 30%) for most attributes, except OM and V, which showed high variability (20% < CV ≤ 30%), and pH, which showed medium variability (10% < CV ≤ 20%). The areas have a pH ranging from acidic to neutral, with low levels (≤2.5%) of OM, very low to very high concentrations (≤10.0 mg dm^−3^ to >60.0 mg dm^−3^) of P, very low to very high concentrations (≤20.0 mg dm^−3^ to >120.0 mg dm^−3^) of K, low to high concentrations (<2.0 cmol_c_ dm^−3^ to >4.0 cmol_c_ dm^−3^) of Ca, and low to medium concentrations (<0.5 cmol_c_ dm^−3^ to >1.0 cmol_c_ dm^−3^) of Mg, with CEC ranging from low to medium (≤7.5 cmol_c_ dm^−3^ to 7.6–15.0 cmol_c_ dm^−3^) [31] and V ranging from very low to high (<45% to >80%) [32]. The low values of pH, Ca, Mg, and V observed in the diagnostic layer (0–20 cm) in most soils in citrus orchards in southern Brazil may occur because the doses of corrective materials were not established following the technical recommendation or the method of application was not adequate [33,34]. Farmers often define doses of corrective materials by considering empirical information, and limestone is applied to the soil surface, without being homogeneously incorporated in the 0–20 cm layer, which makes it difficult for the corrective to descend into the soil profile due to its very low mobility, not being efficient in correcting acidity, and in increasing concentrations of Ca and Mg [33,34]. High concentrations of P and K in the soil may be a consequence of annual applications of doses above the plants’ needs [33,34].

The variability of soil chemical attributes allowed for the fitting of mathematical models using the BL method (Figure 1). These models confirm the correlation of pH and nutrient availability in the soil with citrus fruit yield [35,36]. According to [16], BLs represent the limiting effect of the independent variable on the dependent variable and correspond to the maximum yields that can be attained in each location [15,18]. The data pairs located below the BL correspond to the plots whose yields were reduced by the influence of another independent variable (soil, climate, pests, diseases, nutrients, etc.) or by the interactions of other independent variables [17,37].

Soil pH determines the solubility and availability of nutrients in soil [8]. The adequate pH values for the maximum yield of citrus (5.1–5.6) obtained by the BL method (Table 2) are slightly below the reference pH (6.0) proposed for citrus in the states of RS and SC [31]. However, the soil pH values are within the ideal range (5.0 to 6.5) for citrus growth [38]. It should be noted that citrus are sensitive to acidity and, when grown in soils with a pH lower than 5.0, the greater availability of Al can cause toxicity in their root systems [2]. In areas where acidity is a restrictive factor for citrus growth and development, it is necessary to apply acidity correctives to raise the soil pH and nutrient availability to adequate levels, promoting yield gains and improvements in fruit quality [39,40].

The OM concentrations that promoted the maximum citrus yield (1.0–1.4%) are low (≤2.5%), according to [31]. In this study region, the soils, in general, are highly weathered, with low OM contents [33,34,41]. Therefore, farmers use organic sources of nutrients, such as organic compost [33,34], to increase the availability of nutrients, especially mineral forms of N, which can be absorbed by plants, increasing fruit yield [40,41,42].

The critical and optimal (90 and 100%) P levels in the soil (65.8–129.0 mg dm^−3^) obtained for citrus using BL (Table 2) are higher than those proposed in the regional fertilization recommendation (18.1–36.0 mg dm^−3^ and 30.1–60.0 mg dm^−3^) [31] for soils with clay contents lower than 20% (class 4) and between 21 and 40% (class 3), which predominate in the areas studied. In the case of K, the reference values (161.4–326.0 mg dm^−3^) are higher than those recommended in the regional recommendation (61–120 mg dm^−3^) [31] for soils with a CEC ≤ 7.5 cmol_c_ dm^−3^, which are predominant in the evaluated orchards (Table 1). High concentrations of P and K in the soil have already been reported in the citrus orchards in the main producing regions of southern Brazil and may be related to the frequent and excessive use of industrialized fertilizers and organic residues [33,34]. Excess K in soils can compete with Ca and Mg, inducing a deficiency of these nutrients in leaf tissues, which can cause yield losses [3,14]. Excess P in soils can increase the more labile forms of P, enhancing transfer, especially through the runoff solution, which increases the probability of surface water contamination, making it necessary to quantify the risk of P runoff with current practices [43,44]. The standards for interpreting soil fertility attributes currently used were established for fruit trees in general, considering a very wide region (RS and SC) with great variation in soil and climate conditions, and were not specific to citrus [31]. Divergences in the critical levels of nutrients in the soil and leaf tissues as compared to those in the manuals have also been found in other studies and have been attributed to the use of more demanding cultivars and changes in management [15,18,21].

The adequate concentrations of Ca (0.9–1.4 cmol_c_ dm^−3^) and Mg (0.22–0.34 cmol_c_ dm^−3^) proposed for citrus using the BL (Table 2) method are lower than the adequate concentrations of Ca and Mg (2.0–4.0 cmol_c_ dm^−3^ and 0.5–1.0 cmol_c_ dm^−3^) proposed for fruit trees in general, according to the regional recommendation [31]. These regional recommendation standards were established for a group of plants of different species (fruit trees), and are not specific to citrus fruits, which justifies the differences observed. The lack of pH correction during the implementation of the orchard through liming, associated with the non-homogeneous incorporation of the soil corrective, may be directly related to the lower critical levels of Ca and Mg. However, research results have shown positive correlations between citrus yield and soil pH and exchangeable Ca and Mg concentrations [35,38,40]. This is because an increase in pH contributes to a greater availability of nutrients, and the supply of Ca and Mg, provided by liming, promotes a greater development of the citrus root system, with positive effects on the absorption of other nutrients, plant nutrition, and fruit production [3,36].

The adequate CEC class for citrus (4.5–5.8 cmol_c_ dm^−3^) falls into the low class (≤ 7.5 cmolc dm^−3^), according to the regional fertilization recommendation [31]. These lower critical levels for soil CEC are related to the low values of clay and also of OM in most orchard soils. A positive correlation was even observed between these soil attributes. In general, soils cultivated with citrus in southern Brazil have low OM and CEC values [33,34].

The ideal base saturation (V) for citrus (53.2%) obtained by the BL method (Table 2) is classified as low (45–64%), according to the regional fertilization recommendation [32], and is below the recommendation for citrus (V = 70%) in the state of São Paulo [2]. However, ref. [45], when evaluating the effect of liming (limestone applied on the surface) in a ‘Pêra’ orange orchard in the state of São Paulo, concluded that the maximum production was obtained with a V around 50% and suggested that the base saturation indicated for the crop may be lower than that recommended by the official recommendation.

### 4.2. Relationships Between Leaf Nutrient Contents and Yield

The wide variability observed in soil chemical attributes (Table 2) resulted in a wide variation in yield levels and leaf nutrient contents in the orchards (Table 3). This variability was very high for yield and nutrient contents (CV > 30%), except for N and K, which showed medium variability (10% < CV ≤ 20%), according to [30]. This high variability in yield and nutrient contents in leaf tissues is directly related to the variability of nutrient concentrations in the soil (reflecting fertilization management) and may also be due to the variation in the age of the orchards and monitoring across multiple seasons.

The appropriate ranges of leaf nutrient contents for citrus, established for the producing region of Rio Grande do Sul using the BL method, were compared with those available in the literature (Table 5). It is possible to observe that the ranges vary between the producing regions in Brazil and that, although they are in agreement with those proposed by [4] for the same cultivation region using the Compositional Nutrient Diagnosis (CND) method, they have a smaller amplitude (Table 5). When the contents of these nutrients in the leaves fall below the optimal ranges, it results in yield losses and the need to be corrected through adjustments in the fertilization program [9].

The ranges of adequate contents of N, K, Ca, Mg, and Zn in citrus leaf tissues, obtained using BL, in addition to the lower amplitude, have lower limits than the ranges currently used to interpret the nutritional status of the crops in RS and SC [31]. This pattern was also observed in relation to the ranges proposed for the interpretation of leaf contents in citrus in the state of São Paulo [2]. These lower ranges and with lower amplitudes indicate a lower demand for this micronutrient by citrus [4]. In addition, the doses of these micronutrients based on regional standards [31] are being overestimated. It should be highlighted that, although Cu, Fe, and Mn are required in smaller quantities, their deficiencies impair the photosynthesis of citrus trees, while B and Zn deficiencies affect meristematic growth [2]. It should be noted that the norms established for the south region of the country [31] and for the state of São Paulo [2] were obtained for a wide producing region, encompassing soils and climates that diverged from the current conditions, which justifies the differences.

Foliar and soil analyses are useful in the nutritional management of citrus and should be complementary and never individualized. This is because it is not always possible to establish good correlations between the nutrients in soil and leaf tissues [3,8]. This was observed for P and K, whose critical and optimal levels in the soil (Table 2), despite being much higher than those proposed by the current fertilization recommendation system [31], did not result in high levels of these nutrients in the leaf tissues (Table 4 and Table 5). This can occur because of the low availability of water in the soil, which reduces the probability of nutrients approaching the outer surface of the root, soil compaction, or even ion interactions in the soil, which is very common for P and can decrease the free chemical species of the nutrients in a soil solution [46]. In addition, in the specific case of N, a foliar analysis of this nutrient is used to evaluate its availability in soil and to recommend the doses of nitrogen fertilizer to be applied [10].

The differences observed between the diagnostic norms of soil fertility and plant nutrition between the producing regions reinforce the need to define current and regionalized diagnostic norms for specific climate, soil, and management conditions [3,7,13,14]. It is important that these patterns of interpretation of soil fertility and leaf nutrient content are validated in the field for conditions similar to those in this study [15].

## 5. Conclusions

The soil fertility classes and leaf sufficiency ranges of nutrients were established for citrus-producing regions in southern Brazil, using the BL method. This method made it possible to establish interpretation standards using data from commercial plantations, which present wide variability in soil, climate, and management conditions. These data reflect the best interactions between soil and climate variables and the management of areas that influence productivity, and thus the standards can be more widely applicable. These norms diverged from those used in other citrus-producing regions and reinforce the need to establish regional or local standards to interpret soil fertility and the nutritional status of plants. However, to ensure the accuracy of the established nutritional standards, their validation under field conditions is necessary through fertilization experiments. The norms obtained using the BL method contemplate data from several years and under current cultivation conditions; they will contribute to defining the real need for nutrient application in citrus orchards, greater efficiency in the use of correctives and fertilizers, and lowering environmental impacts.

## Figures and Tables

**Table 1:** Descriptive statistics of soil fertility attributes (0–20 cm) of commercial citrus plots.

Soil Fertility Attributes	Minimum	Maximum	Mean	SD	CV (%)
pH in H_2_O	4.1	7.1	5.5	0.7	12.6
OM (%)	0.7	2.0	1.2	0.3	21.6
P (mg dm^−3^)	1.2	266.6	78.2	61.1	78.1
K (mg dm^−3^)	16.0	590.0	156.6	142.1	90.7
Ca (cmol_c_ dm^−3^)	0.2	4.2	1.7	0.8	47.8
Mg (cmol_c_ dm^−3^)	0.1	0.9	0.4	0.2	39.2
SB (cmol_c_ dm^−3^)	0.5	5.2	2.6	0.9	35.3
CEC (cmol_c_ dm^−3^)	1.7	9.2	5.1	1.6	31.1
V (%)	18.8	84.2	56.5	16.0	28.3

pH in water (1:2.5); OM—organic matter obtained by organic carbon × 1.724 (Walkley–Black); P and K available—Mehlich-1 extractant; Ca and Mg exchangeable—1 mol L^−1^ KCl extractant; SB—sum of exchangeable bases; CEC—cation exchange capacity at pH_7.0_; V—base saturation index; SD—Standard deviation; CV—coefficient of variation.

**Table 2:** Classes for interpretation of soil fertility (0–20 cm) for citrus, established by the boundary line method.

Relative Fruit Yield (RFY)	pH (H_2_O)	OM	P	K	Ca	Fertility Classes
%		%	- - - - - mg dm^−3^ - - - - -		cmol_c_ dm^−3^	
RFY < 70	<4.7	<0.7	<19.6	<40.5	<0.5	Low
70 ≤ RFY < 90	4.7–5.1	0.7–1.0	19.6–65.8	40.5–161.4	0.5–0.9	Medium
90 ≤ RFY ≤ 100	5.1–5.6	1.0–1.4	65.8–129.0	161.4–326.0	0.9–1.4	Adequate ^1^
RFY > 100	>5.6	>1.4	>129.0	>326.0	>1.4	High
**Relative fruit yield (RFY)**	**Mg**	**SB**	**CEC**		**V**	**Fertility** **Classes**
%	- - - - - - - - - - - - - cmol_c_ dm^−3^ - - - - - - - - - - - -				- - - % - - -	
RFY < 70	<0.13	<1.1	<3.5		<31.3	Low
70 ≤ RFY < 90	0.13–0.22	1.1–1.9	3.5–4.5		31.3–40.6	Medium
90 ≤ RFY ≤ 100	0.22–0.34	1.9–2.9	4.5–5.8		40.6–53.2	Adequate ^1^
RFY > 100	>0.34	>2.9	>5.8		>53.2	High

^1^ The lower and upper limits of the range correspond to the critical and optimal levels (90 and 100% of yield); RFY < 70% (FY < 171.4 kg plant^−1^); 70% ≤ RFY < 90% (≤171.4 kg plant^−1^ FY < 220.3 kg plant^−1^); 90% ≤ FY ≤ 100% (220.3 kg plant^−1^ ≤ FY ≤ 244.8 kg plant^−1^); RFY > 100% (FY > 244.8 kg plant^−1^); and pH in water (1:2.5). OM—organic matter obtained by organic carbon × 1.724 (Walkley & Black); P and K—Mehlich-1 extractant; Ca and Mg—1 mol L^−1^ KCl extractant; SB—sum of exchangeable bases; CEC—cation exchange capacity at pH_7.0_; V—base saturation index.

**Table 3:** Descriptive statistics of yield and leaf nutrient contents in citrus.

Nutrients	Minimum	Maximum	Mean	SD	CV (%)
Fruit yield (kg plant^−1^)	6.0	244.8	85.9	47.6	55.4
N (g kg^−1^)	14.9	32.2	22.9	3.1	13.3
P (g kg^−1^)	0.5	2.5	1.3	0.5	34.4
K (g kg^−1^)	4.2	19.6	9.9	3.2	32.0
Ca (g kg^−1^)	13.0	43.9	28.3	5.6	19.8
Mg (g kg^−1^)	0.8	6.5	3.3	1.3	38.3
S (g kg^−1^)	0.7	5.8	3.2	1.1	35.0
B (mg kg^−1^)	13.4	224.1	115.8	48.1	41.5
Cu (mg kg^−1^)	6.8	177.7	58.3	39.4	67.7
Fe (mg kg^−1^)	36.7	243.7	98.4	44.6	45.4
Mn (mg kg^−1^)	7.1	153.6	63.0	31.8	50.4
Zn (mg kg^−1^)	6.6	39.7	17.7	7.6	42.9

SD—Standard deviation; CV—coefficient of variation.

**Table 4:** Leaf sufficiency ranges of nutrients for citrus, established by the boundary line method.

Relative Fruit Yield (RFY)	N		P	K	Ca	Mg	S	Sufficiency Ranges
%	- - - - - - - - - - - - - - - - - - - - - - - - - g kg^−1^ - - - - - - - - - - - - - - - - - - - - - - - - -							
RFY < 70	<16.5		<0.5	<5.2	<15.5	<1.1	<1.2	Low
70 ≤ RFY < 90	16.5–19.1		0.5–0.8	5.2–7.8	15.5–20.9	1.1–2.0	1.2–2.0	Medium
90 ≤ RFY ≤ 100	19.1–22.7		0.8–1.3	7.8–11.3	20.9–28.4	2.0–3.3	2.0–3.0	Adequate ^1^
RFY > 100	>22.7		>1.3	>11.3	>28.4	>3.3	>3.0	High
**Relative fruit yield (RFY)**		**B**	**Cu**	**Fe**		**Mn**	**Zn**	**Sufficiency ranges**
%		- - - - - - - - - - - - - - - - - - - - - - - - - - mg kg^−1^ - - - - - - - - - - - - - - - - - - - - - - - - -						
RFY < 70		<48.3	-	<39.1		<31.1	<7.8	Low
70 ≤ RFY < 90		48.3–88.8	-	39.1–74.3		31.1–55.7	7.8–10.9	Medium
90 ≤ RFY ≤ 100		88.8–127.5	28.3–73.6	74.3–122.5		55.7–89.3	10.9–15.6	Adequate ^1^
RFY > 100		>127.5	>73.6	>122.5		>89.3	>15.6	High

^1^ The lower and upper limits of the range correspond to the critical and optimal levels (90 and 100% of yield); RFY < 70% (FY < 171.4 kg plant^−1^); 70% ≤ RFY < 90% (≤ 171.4 kg plant^−1^ FY < 220.3 kg plant^−1^); 90% ≤ FY ≤ 100% (220.3 kg plant^−1^ ≤ FY ≤ 244.8 kg plant^−1^); and RFY > 100% (FY > 244.8 kg plant^−1^).

**Table 5:** Sufficiency ranges and adequate nutrient contents for citrus, established by the boundary method and those found in the literature.

Reference	N	P	K	Ca	Mg	S
	- - - - - - - - - - - - - - - - - - - - - - - - - - g kg^−1^ - - - - - - - - - - - - - - - - - - - - - - - - - -					
BL-Citrus	19.1–22.7	0.8–1.3	7.8–11.3	20.9–28.4	2.0–3.3	2.0–3.0
[4]	21–26	0.9–1.5	7–11	25–33	1.9–3.7	2.3–3.8
[31]	23–27	1.2–1.6	10–15	35–45	3.0–4.0	-
[2]	25–30	1.2–1.6	12–16	35–50	3.5–5.0	2.0–3.0
**Reference**	**B**	**Cu**	**Fe**		**Mn**	**Zn**
	- - - - - - - - - - - - - - - - - - - - - - - - - - - mg kg^−1^ - - - - - - - - - - - - - - - - - - - - - - -					
BL-Citrus	88.8–127.5	28.3–73.6	74.3–122.5		55.7–89.3	10.9–15.6
[4]	85–149	7–83	50–145		38–94	8–29
[31]	50–100	4.1–10	50–120		35–50	35–50
[2]	75–150	10–20	50–150		35–70	50–75

## Data Availability

Data are contained within the article.
